# Retracted: A Comprehensive Review on Metabolic Syndrome

**DOI:** 10.1155/2019/4301528

**Published:** 2019-01-31

**Authors:** Cardiology Research and Practice

*Cardiology Research and Practice* has retracted the article titled “A Comprehensive Review on Metabolic Syndrome” [1]. The article was found to contain a substantial amount of material from previously published articles (85% of the text), mostly without citation, including the following sources:

- M.-A. Cornier, Dabelea D., Hernandez T. L. et al., “The metabolic syndrome,” *Endocrine Reviews*, vol. 29, no. 7, pp. 777–822, 2008, https://doi.org/10.1210/er.2008-0024 (not cited) [2], mainly in section 5, Pathophysiology, and section 6, Treatment.
- C. Peters, “Metabolic syndrome-a literature review,” Master of Nursing Thesis, University of Arizona, 2007. http://citeseerx.ist.psu.edu/viewdoc/download?doi=10.1.1.503.728&rep=rep1&type=pdf (not cited) [3], mainly in section 5, Pathophysiology, and section 6, Treatment.
- S. M. Grundy, Cleeman J. I., Daniels S. R. et al., “Diagnosis and management of the metabolic syndrome: an american heart association/national heart, lung, and blood institute scientific statement,” *Circulation*, vol. 112, pp. 2735–2752, 2005, https://doi.org/10.1161/CIRCULATIONAHA.105.169404, http://circ.ahajournals.org/content/112/17/2735.full (cited as reference 20) [4], mainly in section 5, Pathophysiology, and section 6, Treatment.
- P. C. Deedwania, R. Gupta, “Management issues in the metabolic syndrome,” *Journal of the Association of Physicians of India*, vol. 54, 2006, http://www.japi.org/october2006/R-797.pdf (not cited) [5], mainly in section 6, Treatment.
- P. L. Huang, “A comprehensive definition for metabolic syndrome,” *Disease Models & Mechanisms*, vol. 2, no. 5-6, pp. 231–237, 2009, doi: https://doi.org/10.1242/dmm.001180, https://www.ncbi.nlm.nih.gov/pmc/articles/PMC2675814/ (not cited) [6], mainly in section 5, Pathophysiology.

This was raised to our attention by the publisher of Cornier et al. Hindawi regrets that this was not identified before publication. The authors said that their aim was to write a comprehensive review extracted from previously published studies on metabolic syndrome and their components, and they apologize for unintentionally not citing most of the sources, but disagree with retraction.
